# The Strain Rate Effects of Coral Sand at Different Relative Densities and Moisture Contents

**DOI:** 10.3390/ma16124217

**Published:** 2023-06-07

**Authors:** Kai Dong, Kun Jiang, Wenjun Ruan

**Affiliations:** School of Energy and Power Engineering, Nanjing University of Science and Technology, Nanjing 210094, China; dongkai@njust.edu.cn (K.D.);

**Keywords:** coral sand, strain rate, moisture content, relative density, volumetric compressive

## Abstract

A 37-mm-diameter split Hopkinson pressure bar (SHPB) apparatus was used for impact loading tests to determine the effects of the relative density and moisture content on the dynamic properties of coral sand. The stress–strain curves in the uniaxial strain compression state were obtained for different relative densities and moisture contents under strain rates between 460 s^−1^ and 900 s^−1^. The results indicated that with an increase in the relative density, the strain rate becomes more insensitive to the stiffness of the coral sand. This was attributed to the variable breakage-energy efficiency at different compactness levels. Water affected the initial stiffening response of the coral sand, and the softening was correlated with the strain rate. Strength softening due to water lubrication was more significant at higher strain rates due to the higher frictional dissipation. The volumetric compressive response of the coral sand was investigated by determining the yielding characteristics. The form of the constitutive model has to be changed to the exponential form, and different stress–strain responses should be considered. We discuss the effects of the relative density and water content on the dynamic mechanical properties of coral sand and clarify the correlation with the strain rate.

## 1. Introduction

Reef flats and lagoons in tropical coastal areas of the world are generally covered by coral sand, which is also called calcareous sand because of its high calcium carbonate content [1]. During the past years, due to the development and utilization of marine resources, many countries have established wharves, airports, oil depots, and other infrastructure on island reefs. Researchers have conducted many studies on the mechanical properties of coral sand as related to engineering needs and the results provided valuable technical support for human exploration and utilization of marine resources [2,3,4,5].

Coral sand is porous and brittle due to a large number of inherent defects in the interior of the particles [6]. It originates from dead coral and shellfish, and is widely used in reef construction. As an engineering material, sand has strong adaptability and should not only be able to support the designed static load but also withstand dynamic loads such as strong shocks, penetrations, and accidental or man-made explosions [7,8]. For a large number of impact engineering problems and accidental emergencies, whether dealing with specific engineering problems or conducting experimental research, the most common three-dimensional stress state of the material is the one-dimensional strain state due to the instantaneousness of the load [9]. Under strong dynamic loads, the mechanical properties of the coral sand have to be determined at a high strain rate (HSR) for safe use in reef development [10].

Coral sand has complex mechanical behaviors under HSR loading and these behaviors differ from those under static loading. Many external factors affect the dynamic mechanical properties of coral sand, such as the relative density, moisture content, particle gradation, and sampling location [11]. These factors, together with the intrinsic strain rate, determine the dynamic mechanical behavior of coral sand. In the moist ocean environment, the water saturation of coral sand and the compactness depend on the location [12,13]. Researchers conducted numerous studies on terrigenous silica bedrock soils and analyzed the influence of compaction and saturation on quartz sand [14,15,16,17,18,19,20,21,22]. However, the mechanical properties of coral sand are substantially different from those of terrigenous sand; thus, the results obtained from quartz sand are not applicable to biogenic coral sand [23,24]. Few studies have been conducted on the HSR characteristics of coral sand. Xiao, Lv, Wu, and Wei investigated the particle breakage characteristics and influence of the moisture content; the study focused on the comparison of coral sand and quartz sand to explain the different dynamic mechanical characteristics attributed to the fragile particles of coral sand [25,26,27,28,29,30]. However, the effects of different strain rates of coral sand have not been comprehensively considered, especially the relationship between the inherent strain rate sensitivity and external natural or artificial conditions, such as compactness and water content.

In this work, one-dimensional strain impact loading tests at different strain rates were performed to determine the dynamic responses of coral sand for different relative densities and moisture contents. Based on previous work [31], the effect of the strain rate and stiffness of coral sand was investigated in detail at different relative densities. In addition, impact loading tests on coral sand with different moisture contents under various strain rates were conducted by a split Hopkinson pressure bar. The effect of the loading rate on partially saturated coral sand was evaluated by determining the mechanism of the softening and yielding response. A novel compressive equation of state (EOS) model describing the relationship between the average pressure and volumetric strain was established by considering different stress–strain responses. The results of this study have great significance for theoretical calculations related to coral reef engineering.

## 2. Introduction of the SHPB Test

### 2.1. Test Device and Test Method

**Figure 1:**
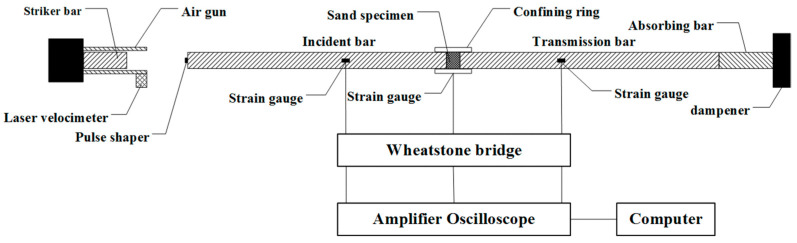
Schematic diagram of modified SHPB.

A dynamic mechanical test was conducted with a Φ37 mm split Hopkinson pressure bar (SHPB) made of aluminum alloy with a density of 2.85 g/cm^3^, an elastic modulus of 72 GPa, and an elastic wave speed of about 5026 m/s. The test device is illustrated in Figure 1. The lengths of the incident bar and the transmission bar are 2000 mm, respectively, and the length of the striker bar is 400 mm. Through the instantaneous release of high-pressure nitrogen, the striker bar is driven to impact the incident bar at high speed to produce a stress pulse, a pulse shaper made of rubber (Φ10 mm × 1 mm, Young’s modulus of 7.83 MPa) is attached to the front face of the incident bar. A sleeve made of high-strength steel (Young’s modulus 210 GPa, Poisson’s ratio 0.29) with an inner diameter of 37 mm (a tolerance of 0~0.01 mm) and an outer diameter of 43 mm is used to restrict the lateral displacement of the sample. A pair of high-precision semiconductor strain gauges are symmetrically attached to the outer walls of the incident bar, the transmission bar, and the sleeve to measure the strain during loading. 

The strain gauge is a semiconductor strain gauge with the type of SB-3.8-120-p-2 made from Avic Zhonghang Electronic Measuring Instruments Co., Ltd. of Hanzhong, Hanzhong, China, with a resistance value of 120 ohms, and the sensitivity coefficient k = 110. The Elsys TranNET FE data acquisition system produced in Switzerland is used for data acquisition, where the sampling frequency is set as 2 MHz, so the data interval obtained is 0.5 μs. The metric signals are transmitted through the Wheatstone bridge and amplifier oscilloscope and converted to voltage signals that are stored in a computer. The strain value at the measured position is calculated by Equation (1) using the parameters of the strain gauge and the amplifier oscilloscope.

(1) $$\varepsilon =\frac{V_{m}}{\zeta \cdot k\cdot V_{a}/\eta }$$

where, $V_{m}$ is the measured voltage signal, ζ is the factor of Wheatstone bridge by using the quarter-bridge, half-bridge and full-bridge, and ζ = 0.25, 0.5, and 1.0, respectively. The half-Wheatstone bridge was used for the tests in this paper, *k* is the sensitivity coefficient of the strain gauge, $V_{a}$ is the input voltage of the Wheatstone bridge, η is the amplification factor of the amplifier in the data acquisition system.

**Figure 2:**
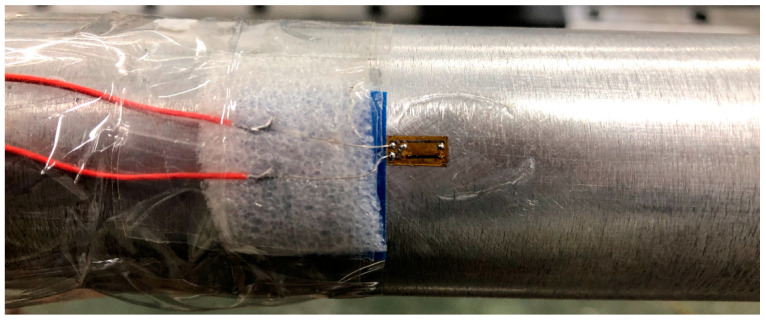
Strain gauge pasting and protection.

In addition, when the stress wave propagates in the bars, the tensile fracture occurs at the connection between the strain gauge and the conductor. As shown in Figure 2, soft foam can be filled between the conductor and the bar, so that the conductor has a flexible buffer when under axial tension, which greatly avoids a conductor fracture.

Assuming that the sample is exposed to uniform stress with uniform deformation during the loading process, the following equation applies: $\varepsilon_{i}(t)+\varepsilon_{r}(t)=\varepsilon_{t}(t)$. According to the one-dimensional stress wave theory, the strain rate $\dot{\varepsilon }(t)$, axial strain $\varepsilon_{x}(t)$ and axial stress $\sigma_{x}(t)$ of the sample during the testing process are obtained using Equation (2) [16,17,28,32].

(2) $$\begin{array}{l} \dot{\varepsilon }(t)=-2\frac{C_{0}}{L_{s}}\varepsilon_{r}(t)\\ \varepsilon_{x}(t)=-2\frac{C_{0}}{L_{s}}\int_{0}^{t}\varepsilon_{r}(t)dt\\ \sigma_{x}(t)=\frac{A_{0}}{A_{s}}E_{0}\varepsilon_{t}(t)=E_{0}\varepsilon_{t}(t) \end{array}\}$$

where $A_{s}$ and $A_{0}$ are the cross-sectional areas of the specimen and bar, respectively, $\varepsilon_{i}(t)$, $\varepsilon_{r}(t)$, and $\varepsilon_{t}(t)$ are the strain of the incident, reflected, and transmitted signals, respectively, $L_{s}$ is the length of the specimen.

When the sample is compressed and deformed under impact loading, the radial expansion is restrained by the elasticity of the sleeve. The circumferential strain of the sleeve is calculated according to the pulse measured by the strain gauge on the outer wall of the sleeve. The pressure on the inner wall of the cylinder $\sigma_{rr}$ and the radial displacement of the inner wall of the sleeve $\varepsilon_{rr}$ are obtained according to the dimensions of the thick-walled cylinder [17,28]. Since the sample is closely attached to the sleeve during compression, based on the interfacial equilibrium condition, the confining pressure and radial strain at the center of the sample are obtained using Equation (3).

(3) $$\left\{ \begin{array}{l} \sigma_{rr}=\sigma_{\theta \theta }=0.5\left(\alpha^{2}-1\right)E_{\mathrm{sl}}\varepsilon_{\mathrm{sl}}\\ \varepsilon_{\mathrm{rr}}=\varepsilon_{\theta \theta }=0.5\left[\left(1-\nu_{\mathrm{sl}}\right)+\left(1+\nu_{\mathrm{sl}}\right)\alpha^{2}\right]\varepsilon_{\mathrm{sl}} \end{array}\right.$$

where α is the ratio of the outer diameter to the inner diameter of the sleeve; $E_{\mathrm{sl}}$, $\nu_{\mathrm{sl}}$, and $\varepsilon_{\mathrm{sl}}$ are Young’s modulus, Poisson’s ratio, and the measured strain of the sleeve, respectively. The three principal stress components of the stress tensor are obtained from the measured axial and circumferential pulses. The average pressure *P* and the volumetric strain $\varepsilon_{v}$ of the sample are defined in Equation (4).

(4) $$P=\frac{1}{3}\left(\sigma_{x}+2\sigma_{\mathrm{rr}}\right),\;\varepsilon_{v}=\varepsilon_{x}+2\varepsilon_{\mathrm{rr}}»\varepsilon_{x}$$

### 2.2. Coral Sand Samples

**Figure 3:**
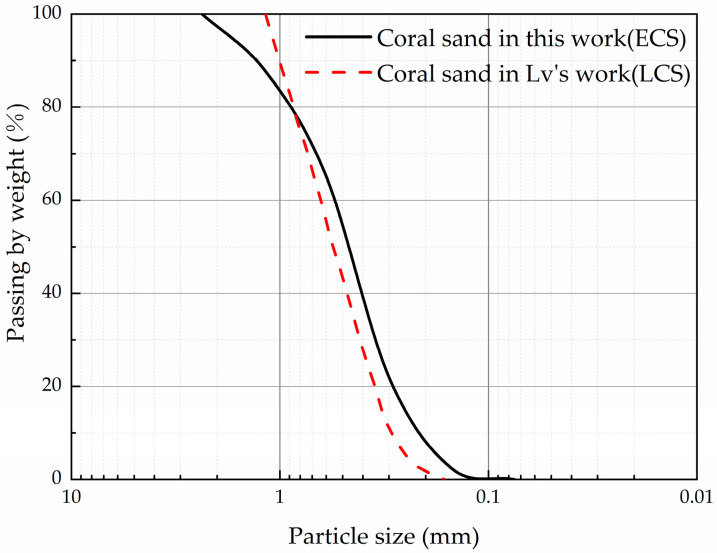
Particle size distribution of the coral sand.

**Figure 4:**
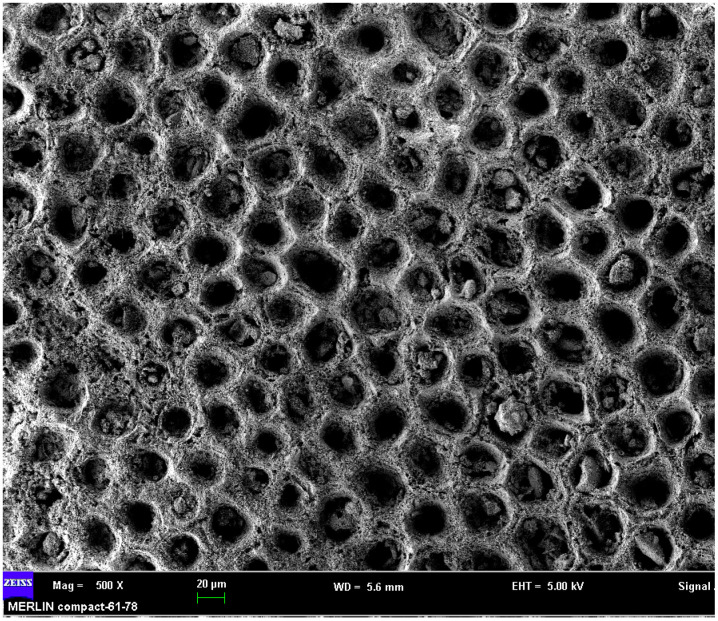
SEM image of the porous coral sand.

The coral sand investigated in this study (ECS) was obtained from the Hainan province in China near the location where the sand used by Lv (LCS) was obtained [28]. The content of CaCO_3_ is over 90%. For the analysis of the mechanical properties, particles with a diameter larger than 2.23 mm and smaller than 0.15 mm were excluded; the mass of these particles was less than 8% of the total. The physical properties of the dry ECS and LCS are shown in Table 1; the ASTM standard was used [33]. The specific gravity of the ECS and LCS is 2.81. The particle size distribution of the sieved sand is shown in Figure 3, and the scanning electron microscopy (SEM) micrographs are shown in Figure 4. The ECS has superior grading than the LCS. 

**Figure 5:**
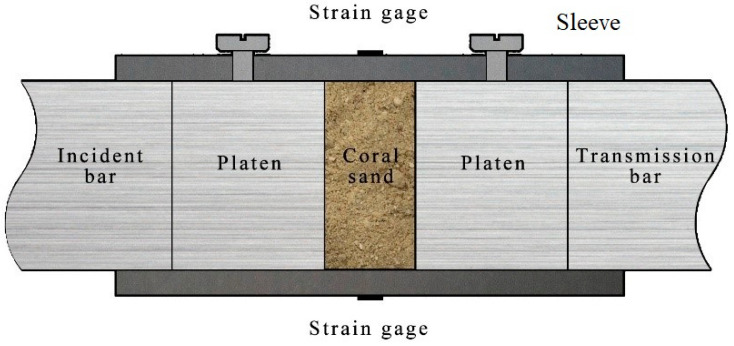
Test section for the coral sand sample.

The preparation of sand specimens has been described in detail in previous studies [19,28,31]. Certain discreteness in the mechanical properties of geotechnical materials requires that the sampling error is strictly controlled during the experiment. First, sieving was conducted prior to sampling using nine different sieve hole sizes and weighing was conducted by particle group using an electronic scale with an accuracy of 0.01 g. This was followed by uniform mixing with a measurement uncertainty of the total mass of ±0.03 g. Second, the length and flatness of the test device were strictly controlled, and the error was within 0.04 mm to ensure the uniform size of the specimens in repeated tests. The test device after assembly is shown in Figure 5. Coral sand is located between two platens which are the same material and diameter as the bar, and have a length of 30 mm. The screws are used to secure the sleeve and SHPB system during assembly, and should be removed during the tests.

After repeated tests and checks, three relative densities were selected, and the relative density Dr can be expressed as Equation (5). The experimental parameters of the specimens are shown in Table 2.

(5) $$\mathrm{Dr}=\frac{\left(\rho_{d}-\rho_{\min}\right)\cdot \rho_{\max}}{\left(\rho_{\max}-\rho_{\min}\right)\cdot \rho_{d}}\cdot 100\%$$

The impact loading tests of the moist coral sand were conducted at a relative density of 60%. The different moisture content conditions are shown in Table 3. The dry density of the coral sand was 1.219 g/cm^3^, and the void ratio *e* was 1.306. The maximum moisture content in this study was 30%, and the maximum saturation was 64.54%. To ensure the uniform distribution of water in the sample, the dry sand is divided into 3–5 parts. Every part is placed in the sleeve and subsequently, a syringe is used to sprinkle the equal division of water into the sand. After 3~5 time repetitions, the thickness of the sample is adjusted using the platens.

## 3. Test Results and Discussion

### 3.1. Pulses and Stress Analysis

**Figure 6:**
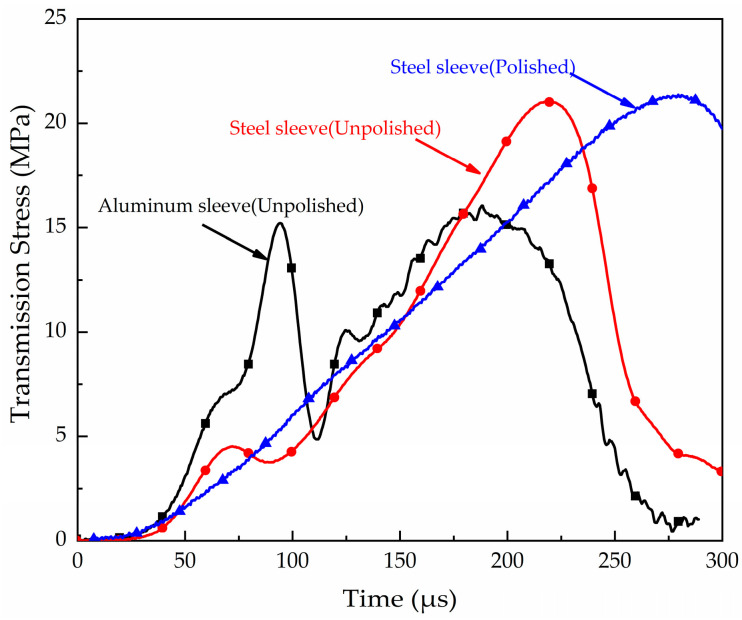
Transmission signals obtained using different sleeves.

In the SHPB test, how to handle the contact fit between the sleeve and the sample, bar, and the platen has a significant influence on the accuracy of the test results. The friction force on the inner wall of the sleeve is a key factor that needs to be avoided. Most scholars use vaseline or lubricating oil to reduce the friction effect of the contact during the test, but many scholars still have a very large initial oscillation in the transmission curve obtained. Martin [16] stated that the vibration of the transmission pulse in the initial stage of loading was a problem that is difficult to explain. This problem can be neglected in high-strength quartz sand but coral sand has low particle strength, and multiple peaks caused by the vibration in the initial stage may result in the misinterpretation of the mechanical properties. Various confining sleeves have been used and shown that the initial vibration may be caused by the asymmetric contact friction between the sleeve and the platen or bar. As shown in Figure 6, the wavy vibration caused by friction is eliminated by ensuring strict processing accuracy, polishing, and grinding of the inner surface of the sleeve, and an application of a thin layer of lubricating oil prior to the experiment.

**Figure 7:**
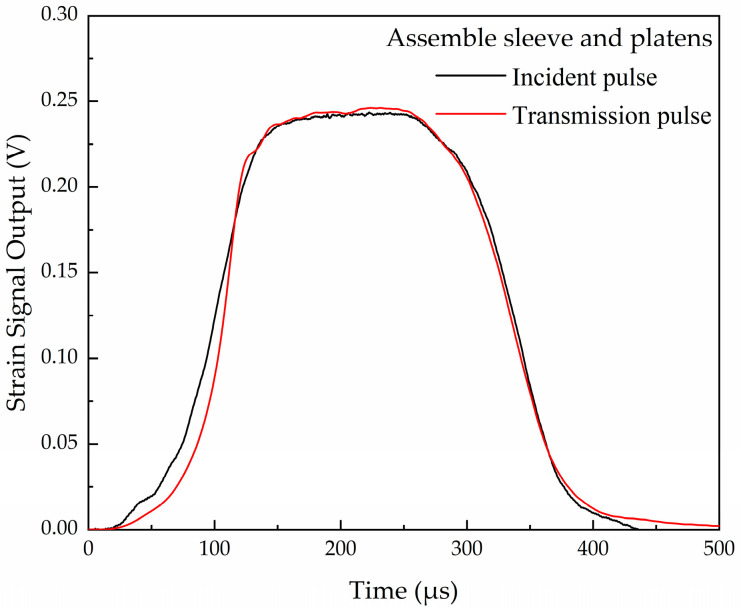
Incident and transmission strain signals without the specimen.

**Figure 8:**
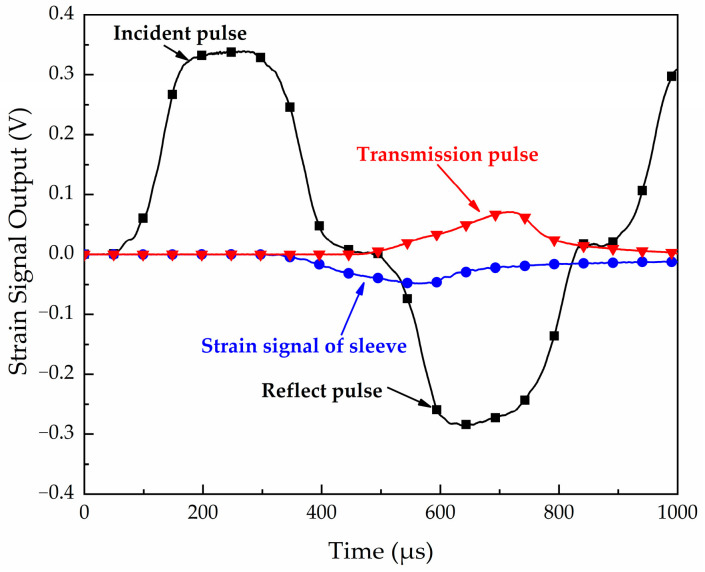
Typical signals recorded from the strain gauges with the specimen.

The sample is connected to the end of the Hopkinson bar through the platen. The incident and transmission strain signals without the specimen (validation test) but assembly sleeve and platens are shown in Figure 7. The results show that the platen and the sleeve have little influence on the test accuracy, and the one-dimensional propagation of the stress pulse is ensured. Typical signals recorded from the strain gauges with the specimen including the incident pulse, reflect pulse, transmission pulse, and strain signal of the sleeve during the test are shown in Figure 8.

**Figure 9:**
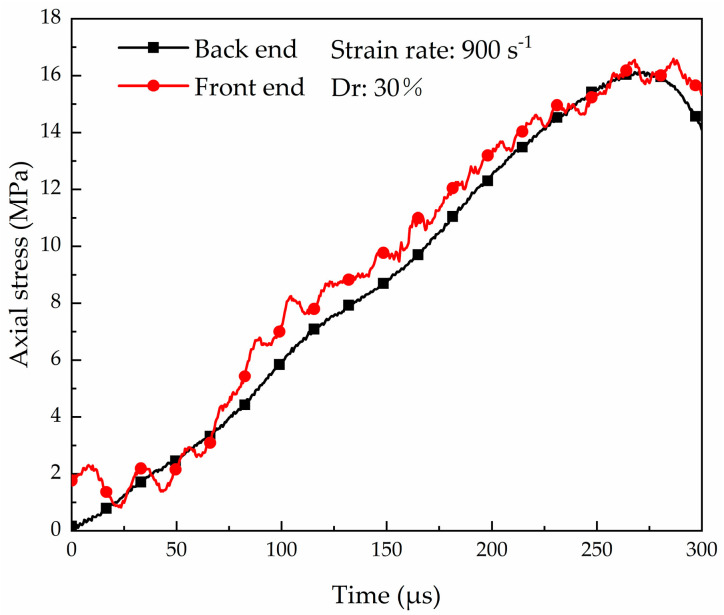
Dynamic stress equilibrium check of the sand sample.

The different strain rates of the specimen are obtained by adjusting the velocity of the striker bar. As the velocity increases, it becomes challenging to ensure uniform loading of the sample under HSR loading. The stress equilibrium at the front and back ends of the sample is the key standard for determining the effectiveness of the test. The back end can be directly measured using transmitted waves of the transmission bar, while the front end requires the use of incident wave subtract reflected measured from the incident bar, i.e., $\sigma_{i}-\sigma_{r}$. The most common method to achieve stress equilibrium is using pulse shaping technology, which increases the rise time of the incident pulse. Figure 9 shows the stress–time history curves of the front and back ends for the HSR and the lowest sample density in the test. It is observed that the stress values are similar at the front and back ends of the sample, indicating that the specimen is under uniform stress during dynamic loading.

**Figure 10:**
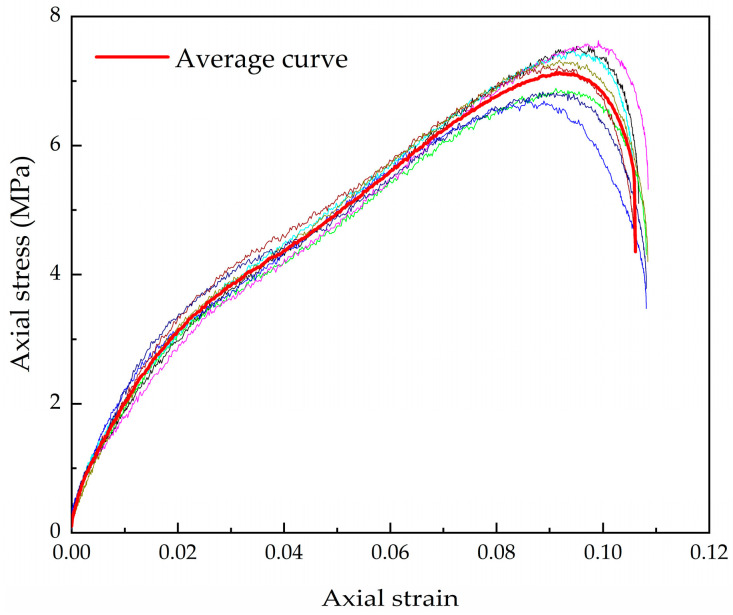
Reproducibility of the axial stress–strain of the test results.

Test reproducibility is an important aspect of geotechnical material testing. The strain pulses recorded by the dynamic strain gauges and the stress–strain curves under the same conditions were obtained using Equation (1). As shown in Figure 10, the consistency of multiple tests demonstrates high reliability and good reproducibility of impact loading. The maximum value of the circumferential strain recorded on the sleeve is in the range of 10^−5^~10^−4^ and the axial strain value of the sample is in the range of 0.08~0.18. This result demonstrates that the sample is in a state of one-dimensional deformation during impact loading. Since the sample deformation is constrained by the steel sleeve, its pressure is very high although the circumferential strain is small and the pressure can be calculated by Equation (2). The larger axial deformation is due to the free compression of the bars on the specimen, which is determined by the impact velocity and specimen properties.

### 3.2. Strain Rate Effected by Compaction of Dry Coral Sand

**Figure 11:**
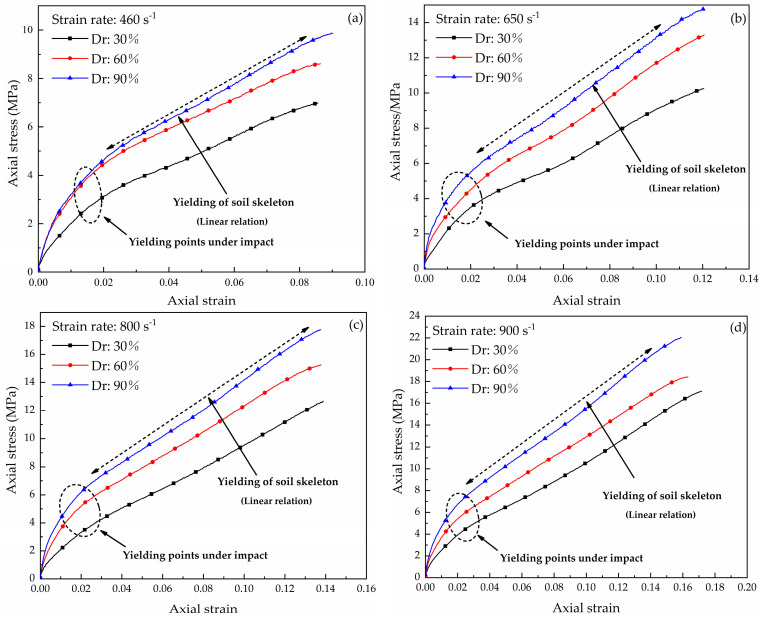
Stress–strain curves of dry coral sand under different compactness: (**a**) 460 s^−1^ (**b**) 650 s^−1^ (**c**) 800 s^−1^ (**d**) 900 s^−1^.

The stress–strain curves of the dry coral sand for the three densities at the strain rates of 460 s^−1^, 650 s^−1^, 800 s^−1^, and 900 s^−1^ were obtained in the literature [31]. The mechanism of the strain rate effect was analyzed, but the relationship between compactness and strain rate was not analyzed in detail in the literature [31]. The curves represent the average of multiple tests (Figure 11). For dynamic compression, the stress–strain responses in this study were significantly different from the experimental results of Lv [28]. An inflection point (yielding point) [10] was observed in the initial deformation stage; this was not observed by Lv. Different yielding characteristics of the stress–strain curves of sand have been reported in the literature, but few scholars have explained the underlying reasons [10]. Lin compared the mechanical properties of Ottawa sand and distinguished two types of responses, i.e., fluid-like and solid-like behaviors; it was concluded that yielding was related to the particle size distribution [20]. The yielding mechanism of coral sand during initial loading is related to the sudden collapse of the specimen skeleton caused by extensive particle breakage. The ECS grading was better than the LCS grading, and the average particle size was smaller; therefore, the ECS particles are more difficult to breakage due to the initial strong skeleton support. When the loading pressure of the specimen exceeded the initial strength (i.e., the yield stress), many were crushed, resulting in a solid mass; therefore, yielding occurred rapidly. However, the LCS particles that were crushed during the entire compression and the curve exhibited fluid-like characteristics.

**Figure 12:**
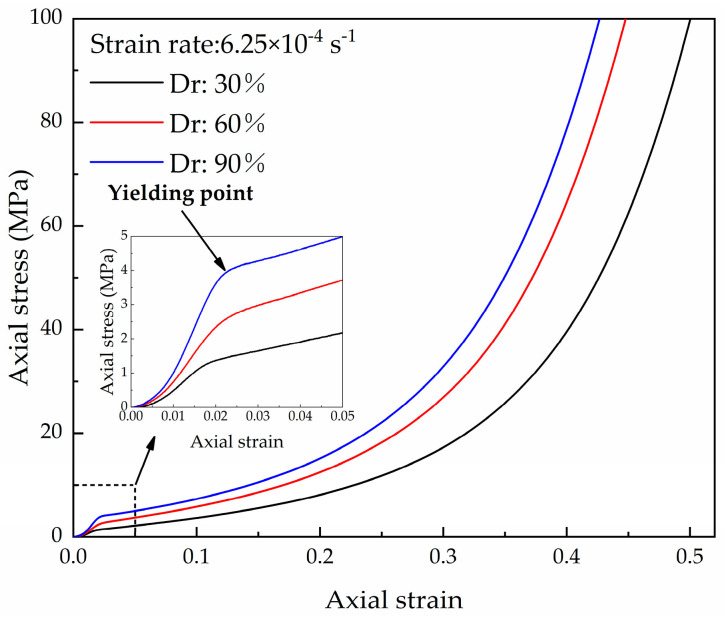
Axial stress–strain curves of dry coral sand under static loading [31].

**Figure 13:**
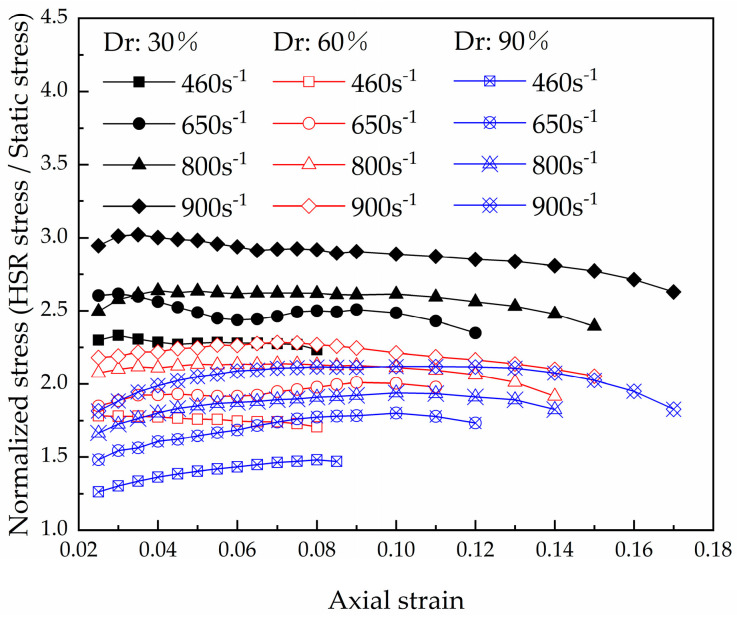
Normalized stress of dry coral sand at different strains in the HSR uniaxial compression tests.

Static compression tests were conducted on the coral sand with the three relative densities using a conventional material test system (MTS) [31]. The yielding points occurred at a strain of 0.02 (Figure 12). This result is similar to the yield strain under HSR loading. With the increase in the compaction level and the increase in the strain rate, the coral sand exhibited increasing stiffness. The dimensionless normalized stress [10] was determined by the ratio of the HRS stress from the dynamic uniaxial compression test and the static stress from a conventional quasi-static test to evaluate the increase in strength due to HSR loading at different strains. As shown in Figure 13, the normalized stress is almost constant during the yielding of the soil skeleton. At the same HSR loading, as the density decreases, the normal stress level increases, indicating that the strain rate sensitivity is closely related to the relative density. This phenomenon is related to the breakage-energy efficiency. A decrease in the compaction level results in higher particle degrees of freedom, thereby increasing the proportion of frictional dissipation [34]. Therefore, the ratio of the crushing energy to the total input energy (i.e., breakage-energy efficiency) decreases. The lower the breakage-energy efficiency during compression, the larger the strain rate effect is [18,20]. This explains why the coral sand shows an increasing strain rate sensitivity when the compaction level decreases.

### 3.3. Effect of Moisture Content on Dynamic Mechanical Properties of Coral Sand

**Figure 14:**
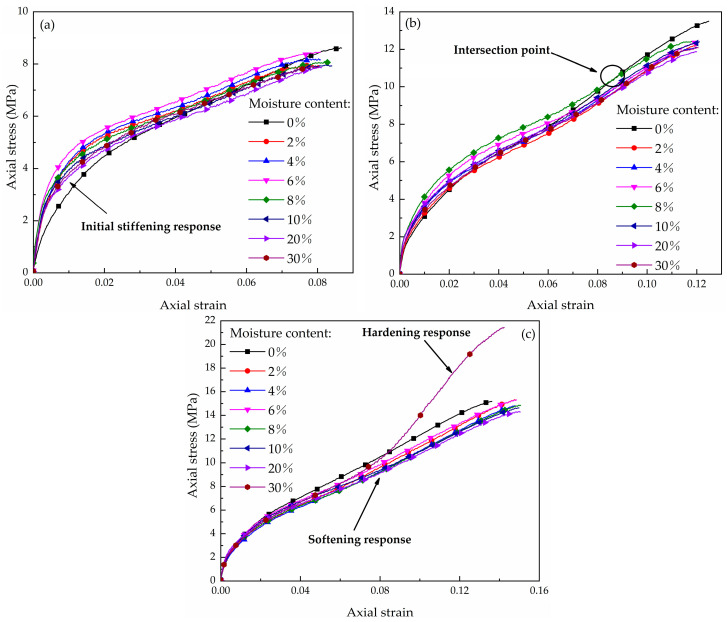
Stress–strain curves of the samples with different moisture contents at different strain rates: (**a**) 460 s^−1^, (**b**) 650 s^−1^, (**c**) 800 s^−1^.

The test results of different moisture contents at strain rates of 460 s^−1^, 650 s^−1^, and 800 s^−1^ are shown in Figure 14. The highest strength of the sample is observed at moisture contents between 6% and 8% and the lowest strength occurs at 20%. The moisture content has little influence on the strength of the coral sand under unsaturated conditions, but some observations regarding the mechanical properties can be made. Generally, the water has a softening effect on the strength of coral sand. Research has shown that water significantly reduces the strength of terrestrial soil, such as quartz sand and clay before reaching a high saturation state [10,16]. However, in this experiment, the influence of the water on the entire strength of the ECS is smaller than that of LCS [29]. Water reduces the friction of particles [10,16,29], and the difference between the responses of the two samples is related to the difference in the frictional dissipation of the particle motion during compression. The frictional dissipation results from the movement of the unbroken particles and the movement of the small sub-particles when the particles are crushed. The ECS has superior grading than the LCS; therefore, there is less particle breakage and the friction dissipation is lower in the ECS than the LCS during compression. As a result, the moist ECS does not decrease significantly due to the lower lubrication efficiency.

The strength of water-bearing sand is higher than that of dry sand during the initial compression process but it is slightly lower than that of dry sand with increasing deformation. This yielding phenomenon is observed at the strain rate of 460 s^−1^ and 650 s^−1^ (Figure 14a,b). These properties are related to the high porosity of coral sand. In this study, the increase in the initial modulus occurs because water is present in the cavities of the particles, resulting in an increase in the strength of the skeleton. However, the water in the supporting pores lubricates the secondary particles as the particles are crushed, causing a decrease in the modulus after yielding.

This effect is also related to the strain rate. It was interesting that when the strain rate increased, the position of the intersection point (where the stress is equal to that of dry sand) moves towards the origin of the coordinates on the abscissa. As shown in Figure 14, the intersection points are located between 0.04 and 0.08 for the strain rate of 460 s^−1^, between 0.02 and 0.07 for 650 s^−1^, and near 0.01 for 800 s^−1^. That is, as the loading rate increases, the initial stiffening response for moist sand occurs at a different location on the curve. This phenomenon is also closely related to the strain rate effect of coral sand. The results in Section 3.2 and those of Huang provide the explanation [18]. As the loading speed increases, the friction dissipation ratio increases, and the water lubrication becomes more effective.

The stress increases sharply at the strain rate of 800 s^−1^ for the initial moisture content of 30%, indicating that the coral sand exhibits a hardening response in the compaction state. The saturation is about 77% upon reaching the compaction point. This demonstrates that coral sand reaches the compaction state earlier than quartz sand or clay, whose saturation is more than 90% [16]. Although there are few inter-particle pores in the hardened section, some unbroken coral sand still has a high internal porosity and does not reach a high saturation state. The strain still increases in the sample due to the release and compression of internal pores caused by particle breakage. However, the compression mechanism in the hardening response is different from that of the non-compaction stage due to the disappearance of the inter-particle pores. In the compaction state, the specimen cannot be easily compressed due to the absence of inter-particle movement. Therefore, the stress increases rapidly with a higher modulus.

**Figure 15:**
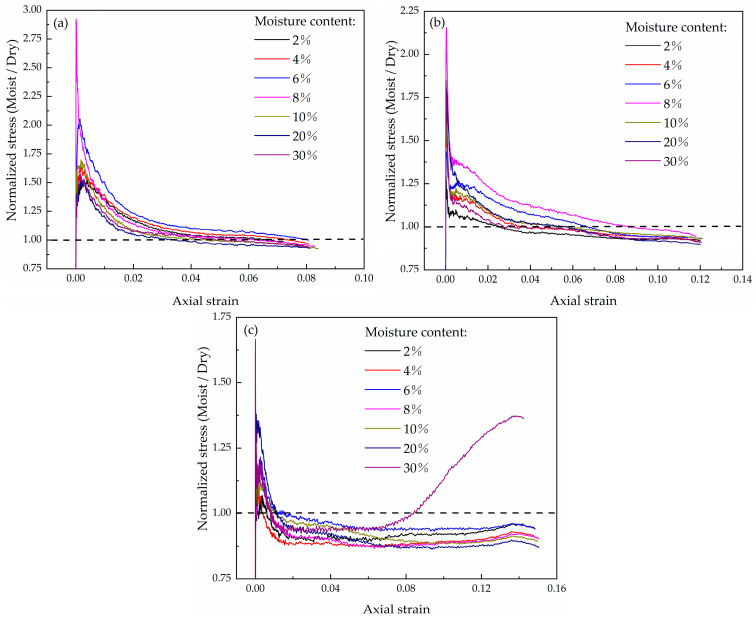
Normalized stress of moist coral sand to dry coral sand: (**a**) 460 s^−1^, (**b**) 650 s^−1^, (**c**) 800 s^−1^.

The research on the mechanical properties of dry coral sand is relatively comprehensive. In order to quantitatively analyze the impact of water content on coral sand, the obtained regular conclusions are applied to previous studies. Based on the dimensionless stress ratio, the relationship between the stress ratio of water containing coral sand and dry sand at different strain rates is established, as shown in Figure 15. It can be more clearly seen that as the strain rate increases, the softening effect of water on coral sand becomes more pronounced.

### 3.4. Effect of Lateral Pressure and Equation of State

**Figure 16:**
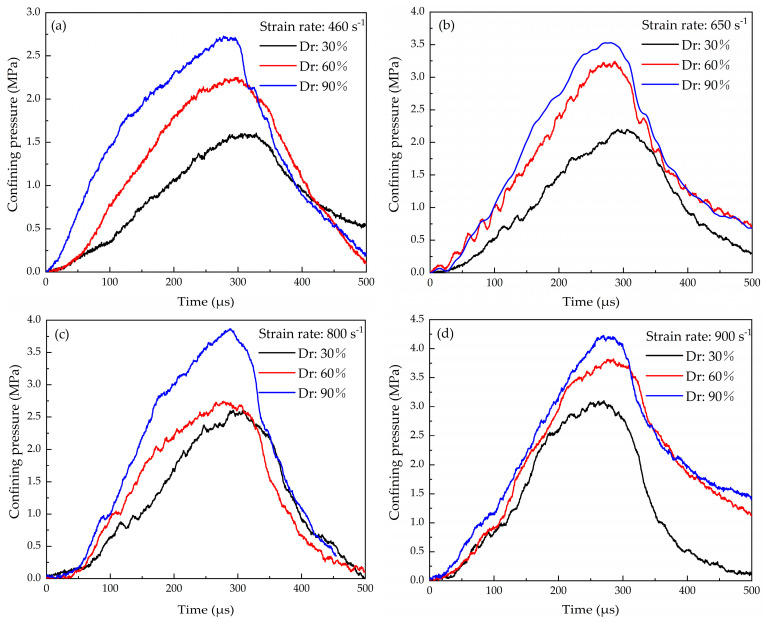
The confining pressure of the coral sand samples at different strain rates: (**a**) 460 s^−1^ (**b**) 650 s^−1^ (**c**) 800 s^−1^ (**d**) 900 s^−1^.

The circumferential strain of the sleeve at strain rates of 460 s^−1^, 650 s^−1^, 800 s^−1^ and 900 s^−1^ was obtained from the pulses recorded at the outer face of the sleeve. The signals were converted into the confining pressure of the sample using Equation (3). As shown in Figure 16, the duration of the pressure increases from zero to the peak value in about 290 μs, which is consistent with the axial loading duration of the sample. The confining pressure increases with the increase in the compaction level at the same strain rate.

**Figure 17:**
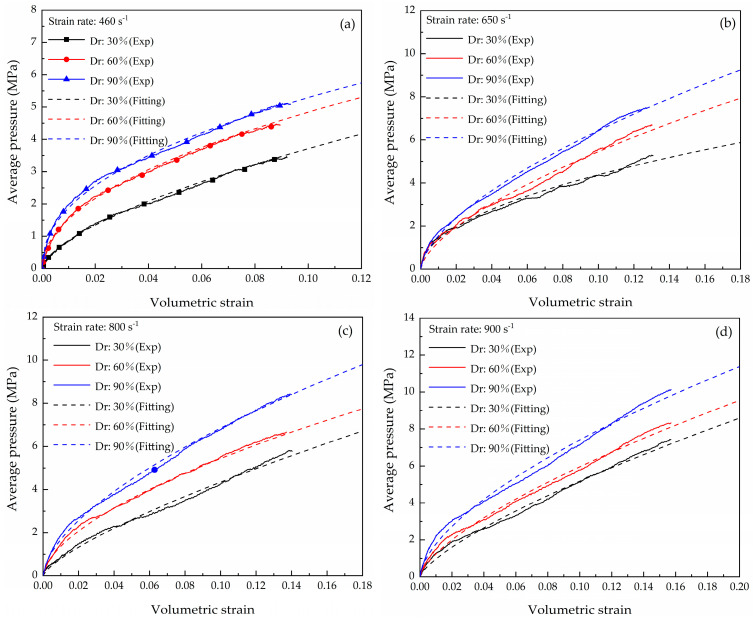
Relationship of average pressure and volumetric strain at different strain rates: (**a**) 460 s^−1^ (**b**) 650 s^−1^ (**c**) 800 s^−1^ (**d**) 900 s^−1^.

The relationship between the average pressure and volumetric strain ($P-\varepsilon_{v}$) of the coral sand at different strain rates is calculated using Equation (4), as shown in Figure 17. However, in this study, in the initial compression stage of the coral sand, the slope of these curves of the coral sand decreases or even remains constant. Therefore, it is necessary to establish the constitutive model and fit the EOS for the solid-like response of the coral sand using exponential form.

The EOS is using the form of $P=a\times \varepsilon_{v}{}^{b}$. The fitting results of the EOS for the three relative densities at different strain rates are shown in Table 4. The value of the goodness of fit $R^{2}>0.95$ indicates that the power exponent form is suitable to describe the solid-like response of coral sand.

## 4. Conclusions

Coral sand has high porosity, irregularly shaped particles, and strain-rate dependency, and exhibits complex mechanical properties. An understanding of the essential mechanical properties allows us to determine the influence of the relative density and the water content on the dynamic mechanical behavior of coral sand, thus providing scientific guidance for practical engineering design and applications. The following conclusions were determined based on the HRS impact experiments of coral sand:

(1) A significant correlation was observed between the strain rate and the stiffness with increasing relative density of coral sand. The breakage-energy efficiency decreases with an increase in the relative density, and the strain rate becomes more insensitive to the stiffness of the coral sand.
(2) The initial stiffening response of the moist coral sand decreased as the loading rate increased. Water had a softening effect on the strength of the coral sand after yielding. Due to the increase in the frictional dissipation of the coral sand with increasing strain rates, the lubrication effect of the water was more noticeable as the strength decreased.
(3) The internal porosity is an important factor affecting the compaction characteristics of coral sand at high saturated moisture content. The hardening state occurred when the inter-particle pores disappeared, and sand breakage became more difficult due to the restricted inter-particle movement.
(4) The compressive response of coral sand should be determined before establishing the pressure-volumetric strain equation. The exponential form of the EOS has to be used for solid-like coral sand.

## Figures and Tables

**Table 1:** Physical properties of the dry coral sand.

Sand Type	D_50_ Particle Size (mm)	Coefficient of Uniformity C_u_	Coefficient of Curvature C_c_	Maximum Dry Density *ρ*_max_ (g/cm^3^)	Minimum Dry Density *ρ*_min_ (g/cm^3^)
ECS	0.48	2.36	0.92	1.317	1.136
LCS	0.55	1.86	0.95	1.377	1.180

**Table 2:** Specimen parameters in the experiments of different densities.

Relative Density Dr	Specimen Quality (g)	Specimen Parameters		Repeated Times
		Density *ρ*_d_ (g/cm^3^)	Thickness (mm)	
30%	15	1.178	11.86	5~8
60%		1.219	11.46	
90%		1.260	11.08	

**Table 3:** Experimental parameters of the specimens with different moisture contents.

Moisture Content	Saturation Degree	Density (g/cm^3^)
0%	0%	1.219
2%	4.30%	1.242
4%	8.61%	1.266
6%	12.91%	1.290
8%	17.21%	1.315
10%	21.51%	1.339
20%	43.03%	1.461
30%	64.54%	1.583

**Table 4:** Fitting parameters of the EOS.

Strain Rate	Dr = 30%		Dr = 60%		Dr = 90%	
	*a*	*b*	*a*	*b*	*a*	*b*
460 s^−1^	15.83	0.63	15.31	0.50	14.92	0.45
650 s^−1^	13.85	0.50	23.80	0.64	26.80	0.62
800 s^−1^	23.86	0.74	21.65	0.60	27.87	0.61
900 s^−1^	27.79	0.73	28.51	0.68	30.86	0.62

## Data Availability

Not applicable.

## Abbreviations

The following abbreviations are used in this manuscript:

HRS	High strain rate
SHPB	Split Hopkinson pressure bar
ECS	Coral sand investigated in this study
LCS	Coral sand used by Lv
MTS	Material test system
EOS	Equation of State
